# Intelligent Fault Diagnosis of Hydraulic System Based on Multiscale One-Dimensional Convolutional Neural Networks with Multiattention Mechanism

**DOI:** 10.3390/s24227267

**Published:** 2024-11-14

**Authors:** Jiacheng Sun, Hua Ding, Ning Li, Xiaochun Sun, Xiaoxin Dong

**Affiliations:** 1 College of Mechanical and Vehicle Engineering, Taiyuan University of Technology, Taiyuan 030024, China; sunjiacheng0801@163.com (J.S.); lining1553604637@163.com (N.L.); 15732651220@163.com (X.S.); dongxxreal@163.com (X.D.); 2 Shanxi Key Laboratory of Fully Mechanized Coal Mining Equipment, Taiyuan University of Technology, Taiyuan 030024, China

**Keywords:** channel attention mechanism, convolutional neural network, fault diagnosis, hydraulic system, multirate data samples

## Abstract

Hydraulic systems are critical components of mechanical equipment, and effective fault diagnosis is essential for minimizing maintenance costs and enhancing system reliability. In practical applications, data from hydraulic systems are collected with varying sampling frequencies, coupled with complex interdependencies within the data, which poses challenges for existing fault diagnosis algorithms. To solve the above problems, this paper proposes an intelligent fault diagnosis of a hydraulic system based on a multiscale one-dimensional convolution neural network with a multiattention mechanism (MA-MS1DCNN). The proposed method first extracts features from multirate data samples using a parallel 1DCNN with different receptive fields. Next, a Hybrid Attention Module (HAM) is proposed, consisting of two submodules: the Correlation Attention Module (CAM) and the Importance Attention Module (IAM), which aim to meticulously and comprehensively model the complex relationships between channel features. Subsequently, to effectively utilize the feature information of different frequencies, the HAM is integrated into the 1DCNN to form the MA-MS1DCNN. Finally, the proposed method is evaluated and experimentally compared using the UCI hydraulic system dataset. The results demonstrate that, compared to existing methods such as Shapelet, MCIFM, and CNNs, the proposed method shows superior diagnostic performance.

## 1. Introduction

The hydraulic system is an efficient transmission device based on fluid mechanics, capable of precise control and powerful output. It has found widespread application in fields such as energy, transportation, and advanced manufacturing. However, hydraulic systems frequently experience failures because of the harsh environments and complex working conditions. If faults in the hydraulic system are not detected and repaired in a timely manner, they may further deteriorate, significantly impacting productivity and endangering production safety [1,2]. Thus, the study of fault diagnosis techniques for hydraulic systems is crucial in terms of enhancing the safety and reliability of mechanical equipment.

With the rapid development of sensor and data transmission technologies, shallow machine learning-based fault diagnosis methods that utilize large amounts of operational data have received increasing attention. For example, Guo et al. [3] proposed a method for identifying the health state of hydraulic systems based on integrated support vector machine. Lei et al. [4] combined principal component analysis (PCA) with extreme gradient boosting (XGBoost) algorithms to enhance the accuracy of fault diagnosis for hydraulic reversing valves. Joo et al. [5] used the Shapelet transform algorithm to achieve fault classification for temporal samples of hydraulic systems. Helwig et al. [6] employed linear discriminant analysis (LDA) to project time-domain and frequency-domain features into a lower-dimensional discriminant space for the predictive maintenance of hydraulic systems. Although fault diagnosis methods based on shallow machine learning have achieved significant results, they are unable to extract features on their own and require manually designed feature extraction algorithms [7]. In addition, shallow machine learning algorithms have limited expressive power to effectively handle complex operating conditions.

As an important branch of machine learning, deep learning methods have been widely used for various tasks, including image recognition [8], object detection [9], and machine translation [10]. The core of deep learning is feature learning. With the powerful modeling ability of deep networks, deep learning can automatically learn complex fault features from data, thus overcoming the shortcomings of shallow machine learning. In recent years, deep learning has gained more and more attention in the field of fault diagnosis [11,12,13]. For example, Wang et al. [14] combined the sliding window spectrum feature with a deep confidence network to realize the fault diagnosis of a hydraulic system. Tang et al. [15] utilized continuous wavelet transform to convert a one-dimensional (1D) pressure signal into a two-dimensional image, which was then input to a 2DCNN for the fault diagnosis of a hydraulic pump. Ji et al. [16] used the Dempster–Shafer theory to integrate the results of the LSTM, CNN, and RF algorithms to improve the diagnostic accuracy of an electromagnetic directional valve. Mallak et al. [17] proposed a structure that combines the LSTM autoencoder and deep learning to realize a fault monitoring and diagnostic method for a hydraulic system in stages. Deep learning algorithms based on a single signal have realized considerable progress in the field of fault diagnosis; however, they are limited to processing the data of a single sensor, which may result in extracted features that lack diversity or comprehensiveness.

Compared to single-sensor data, multisource data can provide more comprehensive information. Fault diagnosis methods based on multisource data will be the trend of the future. Shao et al. [18] proposed a stackable wavelet autoencoder structure with a Morlet wavelet function, which fuses noisy data with vibration data and improves fault diagnostic accuracy. Jing et al. [19] proposed a data fusion method based on a deep CNN that can fuse information from multiple sensors (vibration, noise, and current) adaptively. Wang et al. [20] proposed a method to convert vibration signals from multiple sensors into color images, and Huang et al. [21] designed a multirate data fusion CNN to address the problem where sensors have different sampling rates, and this method achieved fusion of multisource data at the feature level. Wang et al. [22] proposed a heterogeneous ensemble deep neural network (HEDNN) method based on a bagging strategy, which can synthesize the deep features of multisource data to improve the diagnostic accuracy. Buabeng et al. [23] fused flow, pressure, and power data using a systematic combination of hybrid clustering (CLUST) and an optimized multilayer perceptron neural network with unsupervised and supervised learning based on the grey wolf algorithm (GWO-MLP).

The attention mechanism can dynamically adjust focus on different data sources, enhancing the model’s ability to effectively utilize multisource information. Previous studies have designed various attention mechanisms [24,25,26,27] and successfully applied them in the fault diagnosis field. For example, Si et al. [28] introduces the self-attention mechanism into LSTM to achieve high diagnostic accuracy even under variable load and strong noise conditions. Yang et al. [29] presents an attention-parallel multisource data fusion residual network to solve the problems of high similarity of the fault features of different power components cascaded in H-bridge inverters. Song et al. [30] embedded a Convolutional Block Attention Module into a convolutional layer to enhance feature mapping, and good diagnostic results were obtained on limited datasets. Miao et al. [31] proposed an interactive channel attention mechanism to help the fault diagnosis model capture important information from both global and local channels. Fan et al. [32] proposed a multiattention feature fusion network to further enhance the optimization of features by the attention mechanism. Attention mechanisms can enhance the representation of feature information. However, the aforementioned studies merely address the implementation of a single attention mechanism and fail to examine the interactions among various types and scales of attention. Multiattention mechanisms can facilitate the creation of a more refined feature map, which warrants further investigation.

To solve these problems, this study proposes a multiscale 1DCNN with multiattention mechanism for hydraulic system fault diagnosis. The proposed method uses multiple convolutional layers of different scales to extract fault features in sensors with different sampling frequencies. Then, the HAM is employed to enhance the expression of related features. The primary contributions of this study are summarized as follows.

(1) The proposed MA-MS1DCNN can extract deep features from time-series data of varying lengths, enabling end-to-end fault diagnosis. To the best of our knowledge, this is the first application of a multiattention-based multiscale 1DCNN in the fault diagnosis of hydraulic systems.
(2) Two channel attention submodules that focus on different channel information, i.e., the IAM and CAM, are proposed. Further, the HAM is proposed by combining the submodules efficiently using residual connection. The HAM allows the adaptive optimization of features from both importance and relevance perspectives.
(3) Based on a multiscale 1DCNN, this paper designs ablation and arrangement experiments for attention mechanisms, demonstrating that the interaction between different attention mechanisms can effectively improve the accuracy of fault diagnosis. Furthermore, compared to current mainstream methods for hydraulic system fault diagnosis, the proposed approach exhibits stronger diagnostic performance.

## 2. Theoretical Background

### 2.1. 1DCNN

A 1DCNN is a deep learning model that specializes in processing one-dimensional data (e.g., time-series and text). Similar to traditional convolutional neural networks (CNNs), 1DCNNs extract features through convolutional layers. The operation process of the convolutional layer can be expressed as follows:

(1) $$x_{j}^{l}=f(\sum_{i}x_{i}^{l-1}\times \omega_{ij}^{l}+b_{j}^{l})$$

where $x_{j}^{l}$ is the *j*-th output feature of the *l*-th layer, and $x_{i}^{l-1}$ is the *i*-th input feature of the (*l* − 1)-th layer. f is the activation function, $\omega_{ij}^{l}$ denotes the convolution kernel filter that connects the *i*-th input features and the *j*-th output feature map, and $b_{j}^{l}$ is the *j*-th bias of the *l*-th layer.

### 2.2. Residual Connection

**Figure 1:**
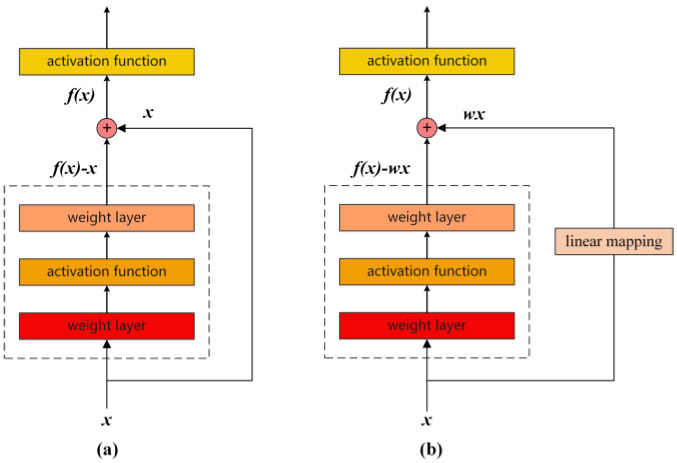
Architecture of (**a**) residual block without linear mapping and (**b**) residual block with linear mapping.

To address the problem of network degradation due to increasing network depth, He et al. [33] proposed a residual network. A residual network introduces shortcut connections by adding the input directly to the output of each residual block, thereby facilitating cross-layer information propagation. The architectures of the residual block are shown in Figure 1.

When the input x has the same dimension as output y, the output of the residual block is given as follows:

(2) y=f(w,x)+x

where f(w,x) represents the residual mapping to be fitted by the residual block. When the inputs x and y have different dimensions, a linear mapping must be added to the residual block to match the dimensions. The residual block output is expressed as follows:

(3) y=f(w,x)+wx

where f(w,x) represents the residual mapping to be fitted by the residual block, and w represents the linear mapping. Shortcut connections provide an additional path for information transfer by adding the input signal to the output signal that has undergone multilayer transformation, thereby effectively mitigating the issue of information fading in deep networks.

### 2.3. Squeeze-and-Excitation Networks

**Figure 2:**
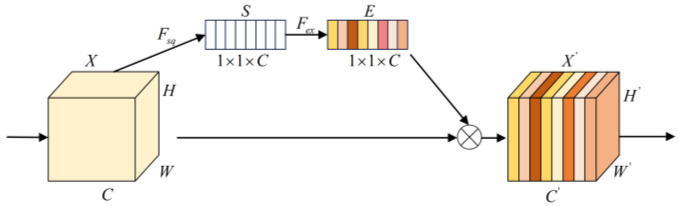
Structure of Squeeze-and-Excitation Networks.

Squeeze-and-Excitation Networks (SENet) are one of the most classical attention mechanisms and consists of two parts, Squeeze and Excitation. The Squeeze part compresses the features using a global average pool (GAP) to obtain statistical features, S, for each channel. The Excitation part excites the statistical features through a fully connected network to produce a set of weight coefficients, E. The structure is shown in Figure 2. The formulas of the above processes are as follows:

(4) $$S=F_{sq}(X)=\frac{1}{H\times W}\sum_{i=1}^{H}\sum_{j=1}^{W}x(i,j)$$

(5) $$E=F_{ex}(S)=\sigma (W_{2}(\delta (W_{1}S)))$$

(6) $$X^{\prime }=X⊗E$$

### 2.4. Efficient Channel Attention Network

**Figure 3:**
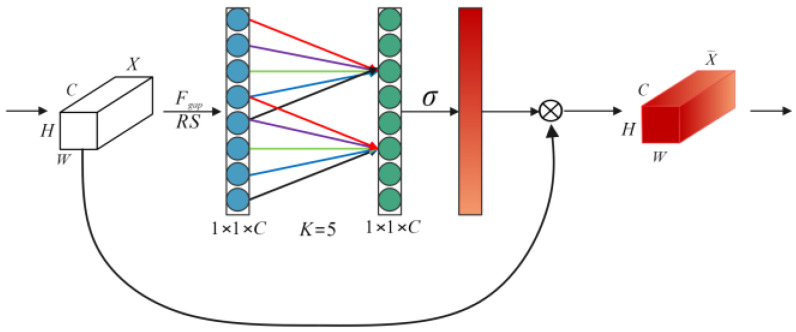
Structure of Efficient Channel Attention Network.

An Efficient Channel Attention Network (ECA-Net) uses convolution to mine the correlation between channels, and its structure is shown in Figure 3. The straight lines in the figure represent convolution operations, and the different colors represent different parameters of the convolution layer. First, the ECA-Net uses a global average pooling layer to compress the input feature map to obtain a 1D feature map containing global information. Then, convolution is employed to capture local cross-channel correlation after reshaping the 1D feature map. Finally, the Sigmoid function is employed to generate the channel weights. The implementation process of ECANet is expressed as follows:

(7) $$\tilde{X}=X⊗\sigma (F_{K}^{1}(RS(F_{gap}(X))))$$

where X is the input feature map, σ is the Sigmoid function, $F_{K}^{1}$ is the convolution layer with a kernel size of K and kernel number of 1, RS represents the reshaping operation, $\tilde{X}$ is the output feature map, and $F_{gap}$ represents the global average pooling operation.

### 2.5. Selective Kernel Networks

**Figure 4:**
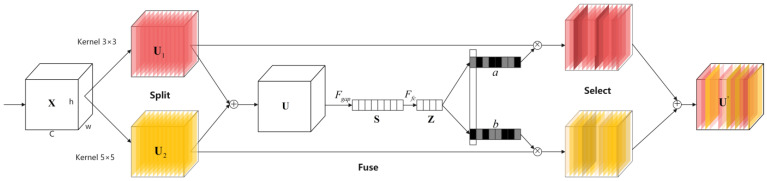
Structure of Selective Kernel Networks.

By fusing multiscale features, networks can effectively utilize information from various scales to create a more comprehensive feature representation. Li et al. [34] proposed Selective Kernel Networks (SKNet), which incorporate a dynamic selection mechanism, allowing these networks to automatically choose different convolutional kernels based on the characteristics of input data, achieving more flexible and efficient feature extraction. The structure of SKNet is shown in Figure 4.

Firstly, two convolution filters with different sizes are used to generate multiscale feature $\bar{U}$.

(8) $$\bar{U}=[U_{1};U_{2}]=[F_{3}^{1}(X);F_{5}^{1}(X)]$$

where $F_{3}^{1}$ represent the convolution layer with a kernel size of 3 and kernel number of 1. $F_{5}^{1}$ represent the convolution layer with a kernel size of 5 and kernel number of 1. The different scale features, $U_{i}$, are fused by element-by-element summation to obtain the fused feature U. Subsequently, U is squeezed and excited to generate the compact feature *Z.*

(9) $$U=U_{1}⊕U_{2}$$

(10) $$Z=F_{fc}(F_{gap}(U))=\delta (\beta (W(S)))$$

where ⊕ represents element-wise summation, $F_{gap}$ is the global average pooling operation, S is the channel-wise statistics, δ is the ReLU function, β represents batch normalization, W represents the linear map, and $F_{fc}$ represents the fully connected layer function. Finally, the weights of each feature scale are calculated using the Softmax function, and then, the weights are weighted and summed with the different scale features, $U_{i}$, to achieve the dynamic fusion of features.

(11) $$a=\frac{e^{Az}}{e^{Az}+e^{Bz}},b=\frac{e^{Bz}}{e^{Az}+e^{Bz}}$$

(12) $$U^{\prime }=a\cdot U_{1}+b\cdot U_{2}$$

where A and B represents the linear map, $U^{\prime }$ is the output feature map, and a and b represent the soft attention vectors for $U_{1}$ and $U_{2}$, respectively.

## 3. Proposed Fault Diagnosis Method

### 3.1. Correlation Attention Module

**Figure 5:**
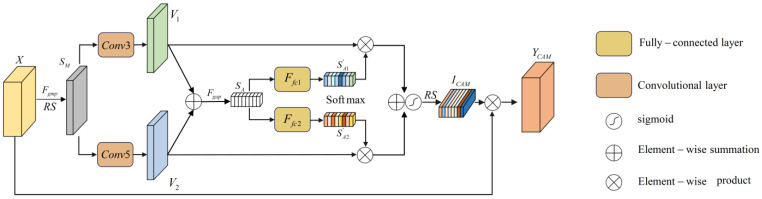
Structure of Correlation Attention Module.

The ECA-Net is limited to the extraction of single-scale correlation and may not improve model performance considerably when handling multisource data with different sampling frequencies. Thus, based on the ECA model, this paper introduces a selective kernel strategy and proposed the Correlation Attention Module (CAM). The CAM can utilize adaptive weights to fuse multiscale correlation to enhance the expression ability of the attention mechanism for complex channel relationships. The CAM primarily comprises three main components, i.e., feature compression, channel correlation construction, and feature recalibration. The basic structure of the CAM is shown in Figure 5. The different stages of the feature maps in the figure are distinguished using different colors, where the attention feature map uses colors with different brightness to express the significant difference in the weights of the feature maps in the channel dimension.

Feature compression: Here, assume that the input to the CAM is $X=[x_{1},x_{2},\ldots ,x_{C}]$, $x_{i}\in R^{W\times 1}$. A global max pooling layer is used to compress the input X to obtain the global feature $S_{M}=[S_{M1},S_{M2},\ldots ,S_{MC}]\in R^{1\times C}$. The *i*-th global feature can be expressed as follows:

(13) $$S_{Mi}=Max[x_{i}(1),x_{i}(2),\ldots ,x_{i}(W)],S_{Mi}\in R^{1\times 1}$$

Then, the feature information $S_{M}$ is reshaped to obtain $S_{M}^{\prime },S_{M}^{\prime }\in R^{C\times 1}$.

Channel correlation construction: First, H convolution filters with different sizes are used to generate multiscale attention maps, $\bar{V}$. In this paper, H is set as 2.

(14) $$\bar{V}=[V_{1};V_{2}]=[F_{3}^{1}(S_{M}^{\prime });F_{5}^{1}(S_{M}^{\prime })]$$

where $F_{3}^{1}$ represents the convolution layer with a kernel size of 3 and kernel number of 1. $F_{5}^{1}$ represents the convolution layer with a kernel size of 5 and kernel number of 1. The different scale attention maps, $V_{i}$, are fused by element-by-element summation to obtain the channel-wise statistics, $V,V\in R^{C\times 1}$. Then, a global average pooling layer is used to compress the statistics, V, and generate a feature map, $S_{A}$.

(15) $$V=V_{1}⊕V_{2}$$

(16) $$S_{A}=F_{gap}(V)=\frac{1}{1\times C}\sum_{j=1}^{C}v_{j}$$

H fully connected layers are used to nonlinearly transform the feature $S_{A}$ to obtain final features, $S_{Ai}^{\prime }$, corresponding to H different scales. Lastly, the Softmax function is utilized to determine the weights, $w_{i}^{\prime \prime }$, for each scale. The process of calculating the weights can be expressed as follows:

(17) $$\begin{array}{c} \left[{S_{A1}}^{\prime },{S_{A2}}^{\prime }]=[F_{fc1}(S_{A}),F_{fc2}(S_{A})\right]\\ =[\delta (\beta (W_{1}(S_{A}))),\delta (\beta (W_{2}(S_{A})))] \end{array}$$

(18) $$w_{i}^{\prime \prime }=\frac{\exp(S_{Ai}^{\prime })}{\sum_{j=1}^{2}\exp(S_{Aj}^{\prime })}$$

where $F_{fci}$ represents the *i*-th fully connected layer function, δ represents the ReLU activation function, β represents batch normalization, and $W_{1}$ and $W_{2}$ represent the linear mapping.

Feature calibration: The different scale attention maps, $V_{i}$, are multiplied by the corresponding weights, $w_{i}^{\prime \prime }$, and then summed to obtain the fusion feature map. Then, the reshaping operation, together with the Sigmoid function, is used to obtain the channel correlation weights, $I_{CAM}$. The input feature map X is multiplied element by element with $I_{CAM}$ to obtain the weighted feature map $Y_{CAM}$. The feature calibration can be expressed as follows:

(19) $$I_{CAM}=RS(\sigma (w_{1}^{\prime \prime }\cdot V_{1}+w_{2}^{\prime \prime }\cdot V_{2}))$$

(20) $$Y_{CAM}=I_{CAM}⊗X$$

where σ represents the Sigmoid activation function, ⊗ represents element-wise product, RS represents the reshaping operation, $w_{1}^{\prime \prime }$ and $w_{2}^{\prime \prime }$ represent the soft attention vectors for $V_{1}$ and $V_{2}$, respectively.

### 3.2. Importance Attention Module

**Figure 6:**
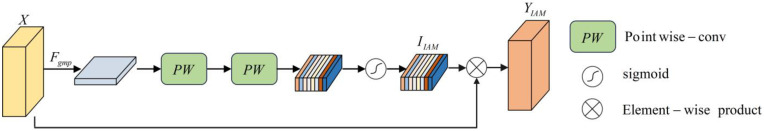
Structure of Importance Attention Module.

The CAM uses convolution to extract features to model the correlation between channels. However, limited by the size of the convolution kernel, the CAM can only focus on locally neighboring channels, making it difficult to effectively express global channel information. Therefore, based on the SENet model, an Importance Attention Module (IAM) is constructed to model the global importance of the channels, and the structure of the IAM is shown in Figure 6.

First, to avoid information loss, two layers of point-wise convolutions with the same channel dimension are used to capture the importance of channels. Then, the attention feature map is processed by the Sigmoid function to obtain the weight $I_{IAM}$. Finally, the output of the IAM, $Y_{IAM}$, is obtained by multiplying $I_{IAM}$ with the input feature map. Assuming that the input feature map of the module is *X*, the above process can be expressed as follows:

(21) $$I_{IAM}=\sigma (F_{1}^{C}(\delta (F_{1}^{C}(F_{gmp}(X)))))$$

(22) $$Y_{IAM}=I_{IAM}⊗X$$

where $F_{1}^{C}$ represents the convolution layer with a kernel size of 1 and kernel number of C, $F_{gmp}$ represents the global max pooling operation, δ represents the Relu activation function, and σ represents the Sigmoid activation function.

### 3.3. Hybrid Attention Module

**Figure 7:**
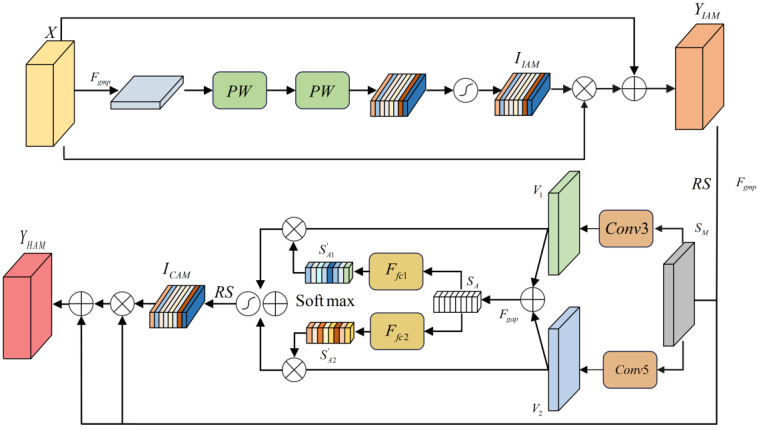
Structure of Hybrid Attention Module.

To fully leverage the intricate information across channels, we devised the optimal fusion of the IAM and CAM, resulting in the introduction of the HAM. The HAM is a combination of the IAM and CAM that integrates the channel importance and correlation information, and it can optimize the input features from different perspectives to further enhance the diagnostic ability of the model. Here, the residual connection concept is introduced to establish a shortcut connection between the input features and the recalibration features, which improves the feasibility of channel information extraction while preserving the original information. The structure of the HAM is shown in Figure 7.

The output of the HAM can be expressed as follows: I have checked I have checked I have checked I have checked

(23) $$Y_{IAM}=I_{IAM}⊗X+X$$

(24) $$Y_{HAM}=I_{CAM}⊗Y_{IAM}+Y_{IAM}$$

where X is the input feature map of the HAM, $I_{IAM}$ is the importance attention weight, $Y_{IAM}$ is the output feature map of the IAM, $I_{CAM}$ is the correlation attention weight, and $Y_{HAM}$ is the output feature map of the HAM.

### 3.4. MA-MS1DCNN

**Figure 8:**
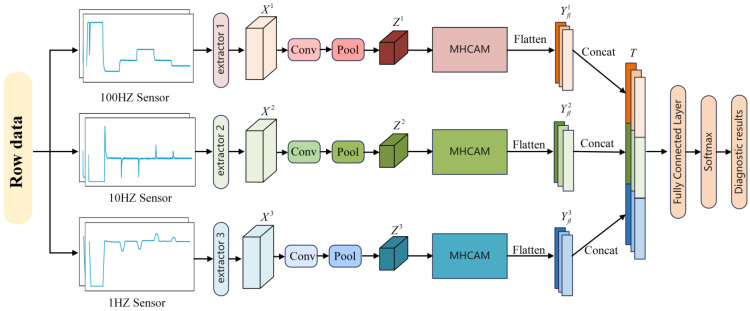
Framework of the proposed method.

The constructed attention module is embedded into a 1DCNN to propose an MA-MS1DCNN. The 1DCNN was selected because the signals collected by the sensors are time-series data represented as one-dimensional structures, which are suitable for input to a 1DCNN. In addition, a 2DCNN typically needs to convert one-dimensional sensor signals (e.g., via wavelet transform and Fourier transform). However, in this conversion process, the sequence and period information of the original signal may be lost. The 1DCNN also has a smaller number of parameters, which facilitates effective real-time diagnosis compared to the 2DCNN. For example, the input layer of the 2DCNN in [13] was 224 × 224 × 3. The MA-MS1DCNN comprises a feature extraction layer, a feature fusion layer, and a fault classification layer, and its overall structure is shown in Figure 8.

The feature extraction layer comprises multiple feature extractors, which are used to extract deep features from raw data with different sampling rates. Each feature extractor contains two convolution blocks comprising a convolution layer, a normalization layer, an activation function layer, and a pooling layer. To suppress high-frequency noise and preserve periodic information, unlike image processing methods that frequently use convolution kernels of size 3 × 3 or 5 × 5, in the first convolutional layer, the MA-MS1DCNN uses a large convolution kernel (e.g., a convolution kernel size of 100 for the 100 Hz feature extractor) to extract the features. Then, the ReLU activation function is applied after the convolutional layer. Through this activation function’s mapping, the MA-MS1DCNN can learn more complex nonlinear features. Finally, the max pooling layer is used to reduce the feature dimension while extracting the most representative features. Here, the input of the *r*-th feature extractor is $X^{r}=[X_{1}^{r},X_{2}^{r},\ldots ,X_{N}^{r}],X_{i}^{r}\in R^{M\times 1}$, where N is the number of sensors at the same sampling rate, and M is the number of samples. After the convolution operation, the timing data are converted into a multichannel feature map $Z=[Z^{1},Z^{1},\ldots ,Z^{r},\ldots ,Z^{R}]$. The *r*-th multichannel feature map is expressed as $Z^{r}=[Z_{1}^{r},Z_{2}^{r},\ldots ,Z_{C}^{r}],Z_{i}^{r}\in R^{W\times 1}$, where W is the length of the feature map and C is the number of feature map channels.

To reduce the redundancy of the features and optimize the extracted features, in the feature fusion layer, the multichannel feature map Z undergoes processing by the HAM, where the channel weights are assigned to recalibrate the multichannel feature map Z into feature $Y=[Y^{1},Y^{2},\ldots ,Y^{r},\ldots ,Y^{R}],Y^{r}\in R^{W\times C}$. This process does not change the size and dimension of the features. Then, feature fusion is performed via flattening and concatenation to output the fused feature map T. Using the attention mechanism to guide multisource data fusion allows the model to utilize important information from multisource data selectively to learn more complex and useful features, which improves the fault diagnosis performance. This process is expressed as follows.

(25) $$Y_{fl}^{r}=F_{flatten}(Y^{r})=[y_{11}^{r},y_{12}^{r},\ldots ,y_{1W}^{r},\ldots ,y_{C1}^{r},y_{C2}^{r},\ldots ,y_{CW}^{r}]$$

(26) $$T=[Y_{fl}^{1},Y_{fl}^{2},\ldots ,Y_{fl}^{r},\ldots ,Y_{fl}^{R}]$$

where $F_{flatten}$ represents the flattening operation, W is the feature length after recalibration, C is the number of feature map channels after recalibration, and $Y_{fl}^{r}$ represents the *r*-th recalibration feature after the flattening operation.

The fault classification layer uses two fully connected layers to reduce the dimensionality of the fusion feature T in a layer-by-layer manner, and the ReLU activation function is implemented to enhance the model’s representation ability. In addition, the Softmax function is used to convert the output of the fully connected layer into the probability of each category of fault.

(27) $$G=F_{fc}(\delta (F_{fc}(T)))$$

(28) $$P_{j}=\frac{\exp(G_{j})}{\sum_{k=1}^{K}\exp(G_{k})}$$

where $P_{j}$ is the probability of category j, $F_{fc}$ is the fully connected layer function, K is the number of fault categories, and G is the Softmax input vector.

## 4. Experiment and Discussion

### 4.1. Experimental Dataset

**Figure 9:**
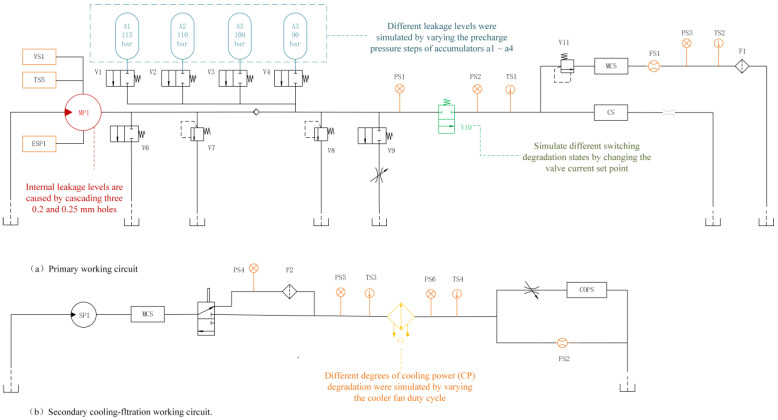
Structure of hydraulic system.

In this study, the hydraulic system dataset from the UCI database was used to verify the performance of the proposed fault diagnosis method. The structure of the hydraulic system is shown in Figure 9, which is briefly described below.

The load level of the hydraulic system is controlled by a (V11) proportional pressure valve. By adjusting the parameters of the V11, the hydraulic system can simulate either a stable working cycle with a constant load or an unstable working cycle with a uniform load change. Here, the working period was fixed to 60 s, and a total of 2205 samples were collected (1449 samples of a steady-state work cycle and 756 samples of an unstable work cycle). To verify the generalizability of the proposed method, this experiment did not differentiate the data of different states. The data were collected from seventeen sensors (fourteen real sensors and three virtual sensors), which were categorized into three different sampling frequencies: seven sensors with a frequency of 100 Hz (including six pressure sensors (PS1–PS6) and one power sensor (ESP1)), two pressure sensors at 10 Hz (FS1–FS2), and eight sensors at 1 Hz (including four temperature sensors (TS1–TS4), one vibration (VS) sensor, one cooling power (CP) sensor, one cooling efficiency (CE) sensor, and one system efficiency (SE) sensor). In addition, failure sample data were acquired by simulating the degradation of several important components (i.e., the hydraulic cooler, pumps, valves, and accumulators). The details of the component degradation are shown in Table 1. Note that component degradation may occur simultaneously, which increases the difficulty of the fault diagnosis process.

### 4.2. Experimental Details

To compare the experimental results, the selection of sensors was consistent with that reported in the literature [20]. Here, three types of sensors with different frequencies were used, including sensors PS1–PS5 (sampling frequency: 100 Hz), FS1 and FS2 (sampling frequency: 10 Hz), and the TS1, VS, CP, and SE sensors (sampling frequency: 1 Hz). The model parameters are shown in Table 2, taking the pump fault diagnosis method as an example. The 2205 samples were randomly disrupted and divided into training and testing sets in a ratio of 8:2. Both training and testing were based on the Pytorch framework in Python 3.9. The hardware environment included a 12th Gen Intel(R) Core(TM) i7-12650H 2.30 GHz processor (Intel, Santa Clara, CA, USA) and an NVIDIA GeForce RTX 4060 GPU (NVIDIA, Santa Clara, CA, USA). Each sample was processed by taking the standard normalization method of subtracting mean and dividing by standard deviation to accelerate the convergence of the model. The Adam optimizer was used during the training process, and the cross-entropy loss function was selected for backpropagation to update the parameters. Here, the learning rate was updated using the learning rate decay approach, which was scaled down to 10% of the original learning rate every 50 iterations. The selection of each hyperparameter was determined based on repeated experiments and manual optimization to obtain the best results. The hyperparameters of the proposed network model are shown in Table 3. It should be noted that the proposed method is robust against parameter selection, and parameter variations have little effect on the final results. The pump fault diagnosis model is used as an experimental object to conduct hyperparameter sensitivity experiments, and the specific results are shown in Table 4.

### 4.3. Feature Visualization Analysis

**Figure 10:**
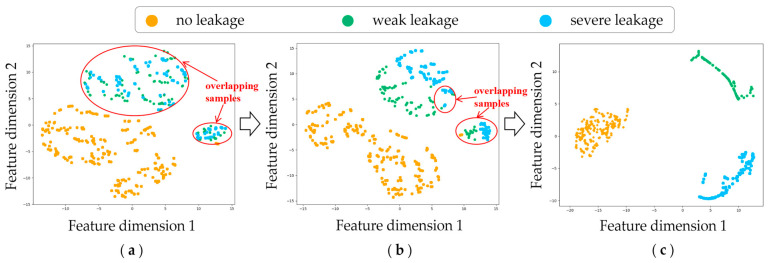
Visualization of the test samples using t-SNE: (**a**) features learned by the feature extractor, (**b**) features optimized by the HAM, and (**c**) features learned by the classification.

To gain an intuitive understanding of the impact of the attention mechanism on the fault diagnosis of hydraulic systems, we selected a hydraulic pump as the analysis subject and employed t-SNE [35] to visualize the feature output at different stages of the model. First, we input the test data to the trained model and extracted feature maps from the various stages of the output. Then, the extracted high-dimensional feature maps were mapped to a 2D plane using t-SNE, where the features were represented as points within a 2D coordinate system. Finally, a scatter plot was generated based on these coordinates. To verify the effectiveness of the HAM, this paper selectively extracts three kinds of features: the features learned by the feature extractor, the features optimized by the HAM, and the features learned by the classification. As shown in Figure 10, after feature extraction, the fault samples with severe leakage were nearly completely separated from the other types of samples, and significant overlap between the samples with weak leakage and no leakage can be observed. After recalibrating the channel weights using the proposed attention mechanism, the number of overlapping samples was reduced significantly, which indicates that introducing the attention mechanism can significantly improve the fault identification performance. The results of the final classifier are shown in Figure 10c. As can be seen, three types of fault samples are completely separated, the clustering boundary is clear, and there is no overlapping phenomenon. These results indicate that the proposed fault diagnosis framework can effectively realize accurate diagnosis of the hydraulic system.

### 4.4. Attention Mechanism and Multiscale Feature Analysis

**Figure 11:**
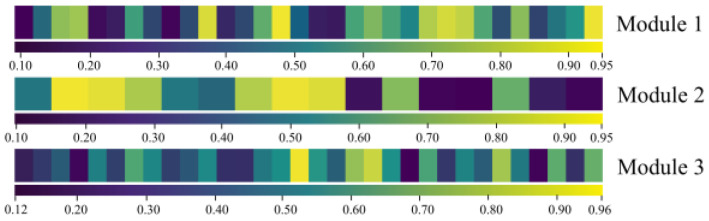
Weight visualization results of three attention modules.

To analyze the proposed attention mechanism, the weight vectors output from the three attention modules in the feature fusion layer are visualized in Figure 11, where the attention weight maps are arranged horizontally in channel order, and the different weights are displayed in different colors. The visualization of the weights reveals the weight values of channels vary from 0.10 to 0.96. These results indicate that the proposed attention mechanism can achieve feature information selection by suppressing irrelevant features. The observations also demonstrate that, even after sensor data filtering, the attention mechanism suppresses information from more than half of the channels, and the weights of approximately one-quarter of the channels were concentrated around 0.1, thereby exhibiting a significant suppression effect. In addition, due to the parameter-sharing nature of the convolution operation, the information between the neighboring channels may be highly correlated. However, the visualization results show significant weight differences, even among adjacent channels, indicating that the proposed mechanism accurately captures channel information and assigns appropriate weights.

Furthermore, the weights $w_{i}^{\prime \prime }$ of different scale features in all CAMs were extracted to analyze the role of the multiscale features in terms of constructing the attention mechanism. Here, to avoid particularity and contingency, 10 experiments were conducted to obtain the average value. The corresponding results are shown in Table 5.

The results demonstrate that the weights of different scale features were roughly distributed in the range of 40–60%. Despite the differences in the importance of different branches, no extreme suppression or enhancement was observed, as in the case of channel attention weights. These findings demonstrate that the channel correlation requires the multiscale feature fusion process to capture more comprehensive information from the features at different scales.

### 4.5. Attention Module Ablation Experiment

Ablation experiments were performed to further verify the effectiveness and necessity of the proposed attention module. The four networks considered in these experiments were structured as follows:

(1) 1DCNN without the attention module;
(2) 1DCNN with the IAM;
(3) 1DCNN with the CAM;
(4) MA-MS1DCNN;

The mean values obtained over 10 ablation experiments are shown in Table 6.

**Figure 12:**
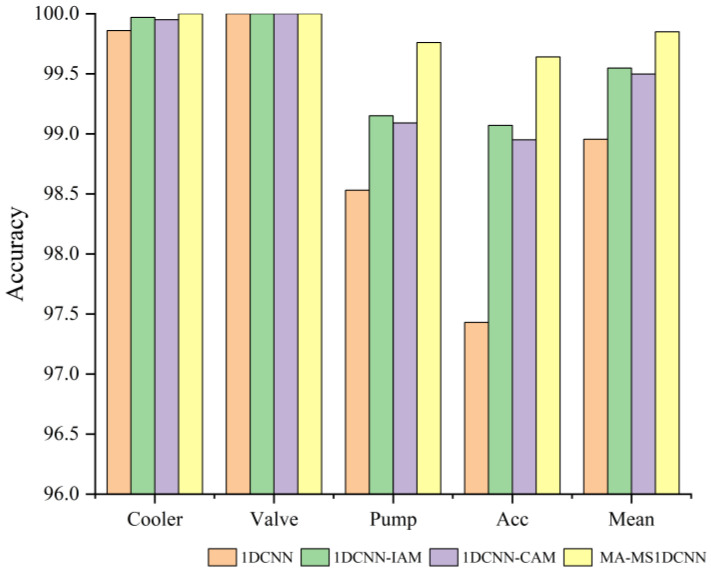
Accuracy of ablation experiments.

Figure 12 shows that the 1DCNN-IAM and 1DCNN-CAM methods obtained better fault diagnosis performance compared to the 1DCNN method, which indicates that the IAM and CAM can optimize the features based on the importance and correlation information of the channels, and this improved the accuracy of fault diagnosis. Note that the MA-MS1DCNN, which integrates two attention modules, exhibited optimal performance in all diagnostic tasks. These results suggest that fusing different levels of information in the process of constructing the channel attention can enrich and deepen the feature characterization and further improve the effectiveness of the feature extraction process.

### 4.6. Arrangement of IAM and CAM

Due to the differences in the functionality of the two submodules, the arrangement may affect the overall performance when they are combined. Thus, three different arrangements of the IAM and CAM were compared in this study, i.e., serial IAM-CAM, serial CAM-IAM, and parallel use of the IAM and CAM. Table 7 summarizes the fault diagnostic accuracy of these distinct arrangements. The results indicate that the accuracy of the serial structure was higher than that of the parallel structure, which may be due to the fact that the direct summing operation in the parallel structure causes the two attention modules to interfere with each other and does not synthesize the multiple units of information between channels effectively. In addition, the performance of the serial IAM-CAM arrangement was better than that of the serial CAM-IAM arrangement. By analyzing the working principle of the submodules, the following conclusion can be obtained. Through the processing of the IAM, the CAM will focus on constructing correlations between important channels, thereby generating a finer attention feature map. Finally, the performance of all three arrangements was better than using any submodule alone, which suggests that synthesizing multiple units of information about the channels is important, and a reasonable arrangement will further enhance performance.

### 4.7. Comparison with Existing Model

To verify the superior performance of the proposed method, it was compared with eight existing methods (the SVM [36], Shapelet [6], ETSC [37], CLUST-GWO-MLP [23], LDA [6], MCIFM [38], CNN [21], and HEDNN [22] methods) on the same experimental dataset. The SVM is a classical shallow machine learning algorithm that is frequently used as a classifier. Shapelet transformation converts time-series data to distance data and then performs fault classification. ETSC is an early classification algorithm specialized for time-series data that enables classification decisions before the time-series data are fully observed. CLUST-GWO-MLP uses unsupervised clustering (CLUST) to group features to reduce complexity and supervised learning via optimized multilayer perceptron neural networks (GWO-MLP) to classify faults. LDA reduces the dimensionality of manually selected high-dimensional features to a low-dimensional discriminant space to realize fault classification. Note that the structures of the CNN and MCIFM methods are similar in that they both adopt a parallel network structure. The difference is that the CNN method uses multiple 1DCNNs with different receptive fields in the feature extraction stage. HEDNNs use a bagging strategy to integrate different DNNs for robust diagnostic results. To reduce the influence of randomness, the above methods were evaluated over 10 repeated experiments, and the average accuracy was considered as the final result. The corresponding results are shown in Table 8.

**Figure 13:**
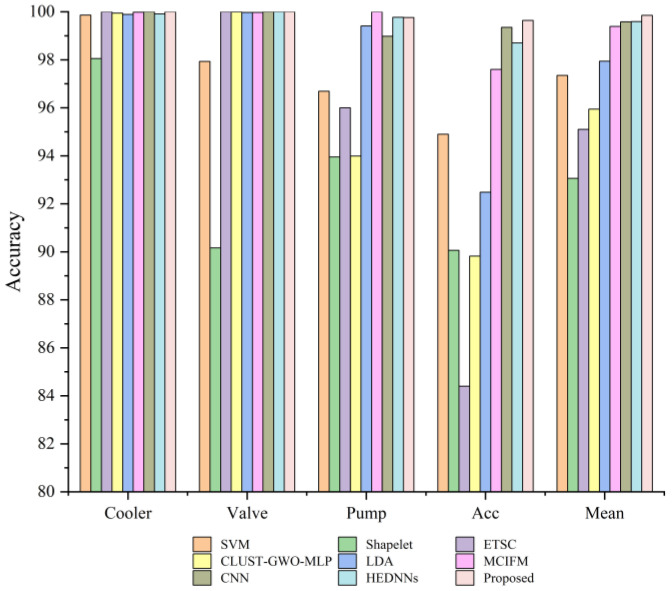
Classification accuracy of different models.

Figure 13 shows that the SVM and ETSC methods, as shallow learning methods, were limited by the difficulty of extracting features and has relatively poor performance. In addition, the feature extraction and model training of the Shapelet, LDA, and CLUST-GWO-MLP methods cannot be optimized jointly, and their performance is not significantly better than that of the SVM and ETSC methods. The MCIFM, CNN, and HEDNN methods involve the powerful feature extraction ability of deep learning, and the diagnostic accuracy was improved considerably. However, all the methods failed to filter the extracted features effectively, and the redundant information will affect the diagnostic accuracy. The proposed method can effectively screen multichannel information and enhance the expression of useful information. Compared to the existing methods, the proposed method achieved excellent results in diagnosing four hydraulic components, showing strong stability. The accuracy for the cooler, valve, and accumulator, as well as the average accuracy across all four components, surpassed that of the compared methods. These findings indicate that the proposed method enables high-precision fault diagnosis for various components in hydraulic systems.

**Figure 14:**
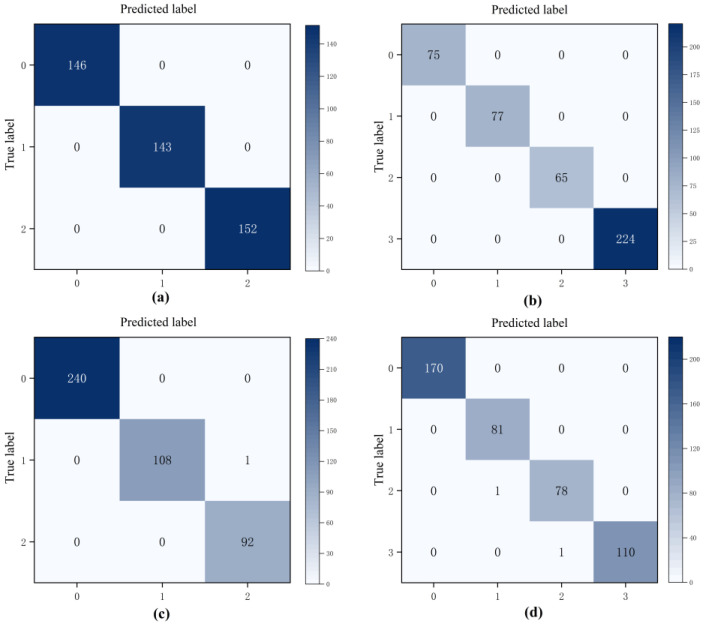
Confusion matrix of the proposed method: (**a**) cooler, (**b**) valve, (**c**) pump, and (**d**) accumulator components.

Figure 14 shows the confusion matrix of the fault diagnosis results for the four hydraulic components. Here, the horizontal axis represents the predicted label of the sample, the vertical axis represents the true label of the sample, and the diagonal of the matrix is the number of samples that were predicted correctly. As can be seen, all samples were classified correctly in the fault diagnosis of the cooler and hydraulic valve components. For the pump and accumulator components, only a few samples were misclassified into adjacent classes, which was primarily caused by the lack of obvious fault characteristics of the sensor signals under continuous degradation.

## 5. Conclusions

This article proposed the MA-MS1DCNN, which introduces an attention mechanism into the fault diagnosis method for hydraulic systems. MA-MS1DCNN employs a multiscale 1DCNN to extract features from raw data at various sampling rates. Subsequently, it develops an HAM that consists of two attention submodules designed to simultaneously capture the importance and relevance information of the channels. This approach enhances the feature selection ability and the fault detection performance of the 1DCNN. The conclusions drawn from this study are as follows: (1) The proposed attention mechanism has demonstrated its effectiveness in enabling the model to comprehend the complex relationships among multisource data, thus allowing for the learning of more salient features. (2) The multiattention mechanism can optimize features from different perspectives, and a judicious combination of these mechanisms maximizes the optimization effect. (3) The efficacy of the proposed method is validated using publicly available hydraulic system datasets, with the experimental results indicating that the diagnostic accuracy of this method in four hydraulic components—the cooler, valve, pump, and accumulator—is maintained at a high level, specifically 100%, 100%, 99.76%, and 99.69%, respectively. In comparison with eight state-of-the-art fault diagnosis methods, the MA-MS1DCNN showed significant advantages in accuracy and stability.

In this study, the attention mechanism was introduced to the fault diagnosis process for hydraulic systems, which further improved the performance of the model. However, there still exist some limitations in the current work, since the CAM and IAM employ a global pooling layer to compress the data in the time dimension when capturing channel significance, which can lead to the loss of some details and local information. In addition, the proposed method does not consider the effect of spatial features on fault diagnosis. Thus, based on the current method, designing an appropriate network structure to utilize the spatial features effectively to improve the accuracy of fault diagnosis will also be the focus of future work.

## Figures and Tables

**Table 1:** Component failure and failure levels.

Component	Fault	Condition	Sample Number
Cooler C1	Cooling power decrease	Full efficiency	732
		Reduced efficiency	732
		Close to total failure	741
Valve V10	Switching degradation	Optimal switching behavior	1125
		Small lag	360
		Severe lag	360
		Close to total failure	360
Pump MP1	Internal leakage	Severe leakage	1221
		Weak leakage	492
		No leakage	492
Accumulators A1–A4	Gas leakage	Optimal pressure	808
		Slightly reduced pressure	399
		Severely reduced pressure	399
		Close to total failure	599

**Table 2:** Parameters in fault diagnosis of the pump.

Extractor	1	2	3
Input length	6000	600	60
Number of sensors	5	2	8
Output channel (Conv1)	16	8	16
Size (Conv1)	100	30	5
Strides (Conv1)	8	1	1
Output channel (Conv2)	32	16	32
Size (Conv2)	15	7	3
Strides (Conv2)	3	1	1
Output nodes (fc1)	32		
Output nodes (fc2)	3		

**Table 3:** Training parameter settings.

Component	Cooler	Valve	Pump	Acc
Optimizer	Adam	Adam	Adam	Adam
Minibatch	64	64	32	32
Epochs	125	125	125	200
Learning rate	0.001	0.001	0.0005	0.0005
Drop factor	0.1	0.1	0.1	0.1
Drop epoch	50	50	50	60

**Table 4:** Results of parameter sensitivity analysis.

Batch Size	Learning Rate			
	0.01	0.001	0.0005	0.00001
16	99.45	99.70	99.73	99.68
32	99.53	99.72	99.76	99.71
64	99.57	99.69	99.72	99.65
128	99.49	99.67	99.70	99.63

**Table 5:** Weights of different branches.

Weight	CAM1	CAM2	CAM3
$w_{1}^{\prime \prime }$	0.52	0.42	0.61
$w_{2}^{\prime \prime }$	0.48	0.58	0.39

**Table 6:** Results of ablation experiments.

Method	Component			
	Cooler	Valve	Pump	Acc
1DCNN	99.86	100	98.53	97.43
1DCNN-IAM	99.97	100	99.15	99.07
1DCNN-CAM	99.95	100	99.09	98.95
MA-MS1DCNN	100	100	99.76	99.69

**Table 7:** Combining methods of IAM and CAM.

Method	Component			
	Cooler	Valve	Pump	Acc
Parallel IAM and CAM	99.98	100	99.23	99.10
Serial CAM-IAM	100	100	99.49	99.41
Serial IAM-CAM	100	100	99.76	99.69

**Table 8:** Performance comparison with existing methods.

Method	Component				
	Cooler	Valve	Pump	Acc	Mean
SVM	99.86	97.93	96.69	94.90	97.35
Shapelet	98.05	90.16	93.95	90.06	93.06
ETSC	100	100	96.00	84.40	95.10
CLUST- GWO-MLP	99.94	99.99	93.99	89.82	95.94
LDA	99.89	99.97	99.41	92.48	97.94
MCIFM	99.98	99.97	100	97.60	99.39
CNN	100	100	98.98	99.35	99.58
DNN	99.91	100	99.77	98.70	99.59
Proposed method	100	100	99.76	99.69	99.86

## Data Availability

The data are publicly available.
